# Derivation and validation of a simple score to predict the presence of bacteria requiring carbapenem treatment in ICU-acquired bloodstream infection and pneumonia: CarbaSCORE

**DOI:** 10.1186/s13756-019-0529-z

**Published:** 2019-05-20

**Authors:** Laura Teysseyre, Cyril Ferdynus, Guillaume Miltgen, Thomas Lair, Thomas Aujoulat, Nathalie Lugagne, Nicolas Allou, Jérôme Allyn

**Affiliations:** 1 Réanimation polyvalente, Centre Hospitalier Universitaire Félix Guyon, La Réunion, Bellepierre, 97405 Saint-Denis cedex, France; 2 Unité de Soutien Méthodologique, Centre Hospitalier Universitaire Félix Guyon, La Réunion, Bellepierre, 97405 Saint-Denis cedex, France; 3 INSERM, CIC 1410, F-97410 Saint-Pierre, France; 4 Laboratoire de bactériologie, Centre Hospitalier Universitaire Félix Guyon, La Réunion, Bellepierre, cedex, 97405 Saint-Denis, France; 5 Comité de Lutte des Infections Nosocomiales, Centre hospitalier universitaire Félix Guyon, La Réunion, Bellepierre, cedex, 97405 Saint-Denis, France; 6 Département d’informatique clinique, Centre hospitalier universitaire Félix Guyon, La Réunion, Bellepierre, cedex, 97405 Saint-Denis, France

**Keywords:** Empiric antibiotic therapy, Health care associated infections, Pneumonia, Bloodstream infection, Carbapenem, Predictive score, Intensive care unit

## Abstract

**Background:**

The recommendations of learned societies mention risk factors for the presence of multidrug resistant bacteria in hospital-acquired infections, but they do not propose a scoring system to guide empiric antibiotic therapy. Our study was aimed at developing a simple score for predicting “the presence of bacteria requiring carbapenem treatment” in ICU-acquired bloodstream infection and pneumonia.

**Methods:**

Between December 2011 and January 2015, we conducted a retrospective study using a prospectively collected French database of nosocomial infections in the polyvalent intensive care unit of a French university hospital. All patients with ICU-acquired bloodstream infection or pneumonia were included in the study. Bivariate and multivariate analyses were performed to develop the CarbaSCORE, and this score was internally validated.

**Results:**

In total, 338 patients were analyzed, including 27 patients requiring carbapenem treatment. The CarbaSCORE was composed of four criteria: “presence of bloodstream infection” (as opposed to pneumonia) scored 2 points, “chronic hemodialysis” scored 4 points, “travel abroad in the last 6 months” scored 5 points, and “MDR-colonization or prior use of a β-lactam of class ≥ 3” scored 6 points. Internal validation by bootstrapping showed an area under the receiver operating characteristic curve of 0.81 [0.73–0.89]. Sensitivity was 96% at the 6-point threshold and specificity was 91% at the 9-point threshold.

**Conclusions:**

The CarbaSCORE is a simple and efficient score for predicting the presence of bacteria requiring carbapenem treatment. Further studies are needed to test this score before it can be used in practice.

**Electronic supplementary material:**

The online version of this article (10.1186/s13756-019-0529-z) contains supplementary material, which is available to authorized users.

## Background

Effective antibiotic therapy is urgent in severe infections, and studies have shown that a delay in effective antibiotic therapy is associated with an increase in the mortality of patients with these infections [1, 2]. For example, a 2008 meta-analysis of 12,296 intensive care patients with ventilator-associated pneumonia (VAP) or blood stream infection (BSI) found a link between delay in effective antibiotic therapy and mortality for both VAP (odd ratio 3.03[1.12–8.19], *p* = 0.03) and BSI (odd ratio 2.28[1.43–3.65], *p* = 0.006) [2]. Other authors have found statistically significant links between delay in effective antibiotic therapy and other outcome variables, such as length of stay in intensive care unit (ICU) and medical management costs [3, 4]. In view of the above, empiric antibiotic therapy must effectively target the bacteria that cause infection.

In contrast to the risk of failure of empiric antibiotic therapy, there is a risk of overuse of broad-spectrum antibiotics, particularly carbapenems. Indeed, the emergence of multidrug-resistant bacteria (MDR) and Extensively Drug-Resistant bacteria (XDR) has become a public health problem [5–11].

The recommendations of learned societies for the antibiotic treatment of hospital-acquired infections mention risk factors for the presence of infection-causing multidrug resistant (MDR) bacteria [12–23]. However, there are discrepancies between these different recommendations—for instance, regarding the presence of a state of septic shock or that of colonization with MDR bacteria. Moreover, these various risk factors have not been studied together, which raises the possibility of redundancies and multicollinearities [24]. Lastly, no score has been recommended to guide prescribing physicians in the choice of empiric antibiotic therapy for ICU-acquired infections.

The aim of this study was to develop a simple score for predicting “the presence of bacteria requiring carbapenem treatment” in ICU-acquired bloodstream infection and pneumonia.

## Methods

### Study population

Between December 2011 and January 2015, we conducted a retrospective study using a prospectively collected French database of nosocomial infections in the 23-bed medical/surgical adult ICU of a French university hospital (Centre Hospitalo-Universitaire Félix Guyon, Saint-Denis). The study was approved by the Ethics Committee of the Centre Hospitalo-Universitaire de La Réunion (reference R15016). We included all patients with ICU-acquired bloodstream infection or pneumonia that occurred more than 2 days after admission and that were reported to the national network for the surveillance of ICU-acquired infections (REA-raisin network). Excluded from the analysis were patients under 18 years of age, patients with non-bacterial or undocumented infection, and patients whose incomplete records did not allow for analysis. Only the first episode of ICU-acquired infection was analyzed.

### Data collection and processing

We analyzed demographic data, status on admission, microbiological examination results, severity scores at the time of infection, modified Clinical Pulmonary Infection Score (CPIS), therapies performed, and patient outcome (death, duration of ventilation, and length of stay in ICU) [25, 26]. In the studied ICU, screening for the risk factors for infection with MDR bacteria mentioned in the 2004 guidelines of the French Society of Anesthesia and Intensive Care Medicine (SFAR) and the 2005 guidelines of the American Thoracic Society (ATS) is performed routinely [18, 21]. These risk factors are as follows: antibiotic therapy in the previous 90 days, travel abroad in the last 6 months, hospitalization/homecare/institutionalization in long-term care or in residential care for dependent elderly people, chronic hemodialysis, long-term invasive device (tracheostomy, gastrostomy, indwelling urinary catheter), hospitalization for at least 2 days in the last 6 months, immunosuppression, and colonization with MDR bacteria.

In the studied ICU:

- All patients undergo screening for rectal colonization with MDR or extensively drug-resistant (XDR) bacteria (see definitions below). Rectal swabs were collected upon admission and then once a week. Swabs containing transport medium (Amies agar gel medium transport, Copan, USA) were transferred into infusion for 24 h. Then 10 μL of broth was on streaked onto (i) ChromID ESBL agar (Biomerieux, Marcy-l’Etoile, France) as recommended by the French Council of Public Health and (ii) Drigalski agar (Biorad, Marne-la-Coquette, France) with antibiotics disks (Biorad, Marne-la-Coquette, France) added to detect resistant strains. Agar plates were incubated for 24 h to 48 h at 35 °C. Bacterial identifications were performed using the Walk Away system (Siemens, USA) or gallery API20E or 20NE (Biomerieux, Marcy l’étoile, France). Susceptibility testing was systematically obtained to confirm the resistance mechanism following French recommendations. Only rectal swabs containing faeces were analyzed.
- No other samples are taken for the surveillance of colonization, including at the pulmonary level.
- All other microbiological samples are taken only in case of suspicion of infection, with the aim of identifying the infection-causing bacteria.
- All bloodstream infection and pneumonia occurring more than 2 days after ICU admission are reported to the REA-raisin network.

The selected date of infection was the date of microbiological sampling (blood culture or lung sample). The primary endpoint was “the presence of bacteria requiring carbapenem treatment,” as defined by antimicrobial susceptibility testing analysis of the infection-causing bacteria. Antimicrobial susceptibility testing analysis was performed by one of the investigators (LT).

### Definitions

Multidrug resistant bacteria included extended-spectrum β-lactamase-producing Enterobacteriaceae (ESBL-E) strains, ceftazidime-resistant *Pseudomonas aeruginosa* and *Acinetobacter baumannii* strains, carbapenem-resistant *Pseudomonas aeruginosa* strains, and methicillin-resistant *Staphylococcus aureus* (MRSA) strains [27].

Extensively drug-resistant bacteria included carbapenemase-producing Enterobacteriaceae (CPE) strains, carbapenem-resistant *Acinetobacter baumannii* strains and glycopeptide-resistant enterococci (GRE) strains [28].

Colonization with MDR/XDR at the time of ICU-acquired infection was defined by knowledge of one positive hygiene or clinical sample for MDR/XDR at least 2 days before the date of infection.

Following recommendations of the French Society of Anesthesia and Intensive Care Medicine (SFAR) and the French Institute for Health Surveillance (InVs), ICU-acquired pneumonia was defined by: radiological signs with two successive images suggesting a focus of pneumonia (a single image being sufficient in the absence of a history of heart disease or of an underlying lung disease), and at least one of the following signs: temperature > 38.3 °C with no other cause, leukocyte concentration < 4 G/L or ≥ 12 G/L, and at least two of the following signs: purulent tracheobronchial secretions, cough or dyspnea, and oxygen desaturation or increased oxygen requirement or ventilation demand [20, 29].

The diagnosis had to be confirmed quantitatively by deep respiratory sampling: bronchoalveolar lavage with a positivity threshold ≥10^4^ colony-forming units (CFU)/ml or protected distal sampling with a positivity threshold ≥10^3^ CFU/ml or, failing this, tracheobronchial aspiration with a positivity threshold ≥10^6^ CFU/ml.

ICU-acquired bloodstream infection was defined by the presence of at least one positive blood culture—with the exception of coagulase-negative *Staphylococci* and other saprophytic microorganisms, for which two positive blood cultures were required.

Following the classification proposed by Weiss et al., β-lactams were ranked into six classes according to spectrum of activity (Additional file 1: Table S1) [30].

“The presence of bacteria requiring carbapenem treatment” was defined by the identification of one or more bacterial strains requiring treatment with a β-lactam class 6, with no possibility of using a lower class of β-lactams.

Carbapenem overuse was defined by empiric treatment with a β-lactam class 5 or 6, with the possibility of using a lower class of β-lactams based on antimicrobial susceptibility testing results.

Carbapenem treatment failure was defined by one (or more) bacteria causing infection was resistant to carbapenem (class 6), according to the antimicrobial susceptibility testing.

### Statistical analysis

Results were expressed as medians and interquartile ranges for quantitative variables and as frequencies and proportions for qualitative variables. Quantitative variables were compared using the Mann-Whitney test. Qualitative variables were compared using the Pearson Chi-square test or Fisher’s exact test, as appropriate. All analyses were performed with a bilateral alpha risk of 5%.

Variables collected at the time of empiric prescription of antibiotic therapy that were statistically associated in bivariate analysis with “the presence of bacteria requiring carbapenem treatment” (with a *p* <  0.05) were entered into a backward stepwise logistic regression model. A score was then developed from the model obtained by regression analysis, using as weight the logistic regression coefficient rounded to the nearest whole number. The calibration of the model was evaluated using the Hosmer-Lemeshow test, and the Nagelkerke and Cox/Snell R^2^ were calculated. Score performance was measured using the area under the receiver operating characteristic (ROC) curve and its 95% Confidence Interval. Finally, score performance was validated by bootstrapping (2000 replicates). The software SPSS version 15 (SPSS Inc., Chicago, Ill, USA) and R (pROC package) were used for data analysis [31–33]. This article follow the STROBE reporting guidelines for observational studies.

## Results

**Fig. 1:**
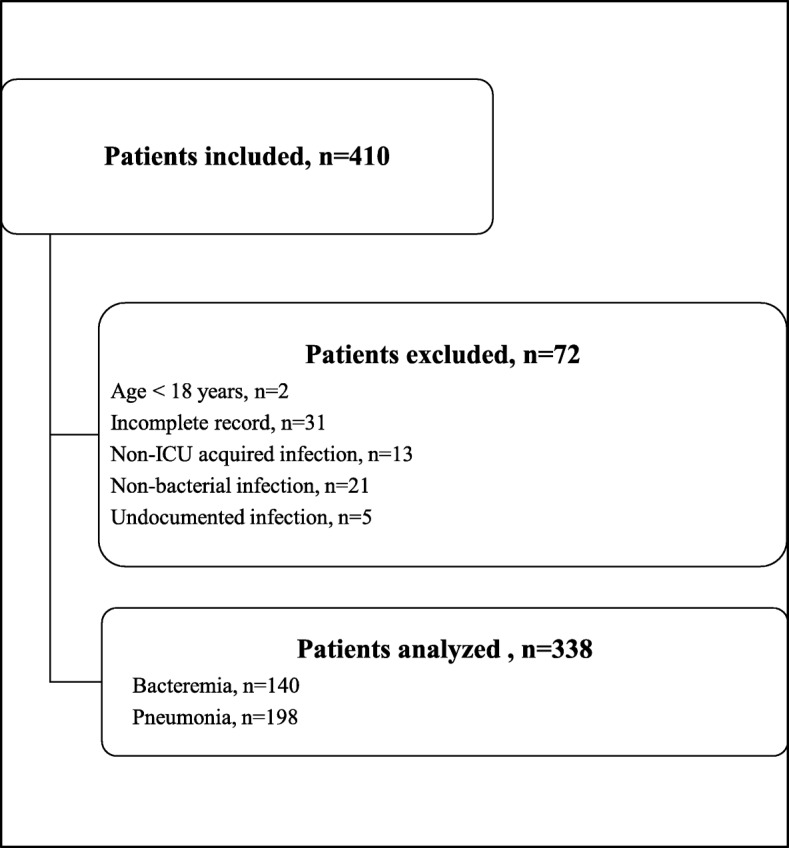
Flowchart

Over the 37-month study period, 2584 patients were admitted to the ICU. Among these, 410 (15.8%) presented at least one ICU-acquired bloodstream infection or pneumonia more than 2 days after ICU admission, and were consequently included in the study. Seventy-two of these patients were excluded from the analysis, and the remaining 338 patients formed the study cohort (Fig. 1).

### Characteristics of patients at ICU admission

The characteristics of the 338 analyzed patients are presented in Table 1. Antimicrobial susceptibility testing of the bacterial strain responsible for infection in the intensive care unit indicates that carbapenem treatment was required in 27 cases (8%). The variables significantly associated with the variable “presence of bacteria requiring carbapenem treatment” were:

- Chronic renal failure (*p* = 0.03) and chronic hemodialysis (*p* = 0.01)
- Travel abroad in the last 6 months (*p* <  0.001)
- Colonization with MDR/XDR known on ICU admission (*p* = 0.02) or 2 days before the date of nosocomial infection (*p* < 0.001)
- Prior use of a β-lactam of class ≥3 (*p* = 0.02)
- A delay of more than 7 days between ICU admission and date of nosocomial infection (*p* = 0.05)
- Type of ICU-acquired infection (bloodstream infection as opposed to pneumonia, *p* = 0.02)

**Table 1:** Sociodemographic, clinical-biological, therapeutic, and outcome characteristics of patients

Variable	Total (*n* = 338)	Carbapenem not necessary (*n* = 311)	Carbapenem necessary (*n* = 27)	*p*
Age (years)	61 [49–71]	59 [19–87]	61 [22–83]	0.21
Male sex, n (%)	243 (71.9)	224 (72.0)	19 (70.4)	0.83
Chronic diseases, n (%)				
• High blood pressure	186 (55.0)	168 (54.0)	18 (66.7)	0.23
• Coronary artery disease	85 (25.1)	77 (24.8)	8 (29.6)	0.64
• Arteriopathy	59 (17.5)	53 (17.0)	6 (22.2)	0.44
• Diabetes	117 (34.6)	110 (35.4)	7 (25.9)	0.40
• Chronic heart failure	64 (18.9)	57 (18.3)	7 (25.9)	0.31
• Chronic respiratory failure	46 (13.6)	43 (13.8)	3 (11.1)	1.00
• Chronic obstructive bronchitis	46 (13.6)	43 (13.8)	3 (11.1)	1.00
• Cancer, immunosuppression^a^	59 (17.5)	53 (17.0)	6 (22.2)	0.44
• Chronic alcoholism	98 (29.0)	90 (28.9)	8 (29.6)	1.00
• Chronic tobacco exposure	91 (26.9)	85 (27.3)	6 (22.2)	0.66
• Obesity	59 (17.5)	53 (17.0)	6 (22.2)	0.44
• Undernutrition	47 (13.9)	44 (14.1)	3 (11.1)	1.00
• Chronic renal failure^b^	55 (16.3)	46 (14.8)	9 (33.3)	0.03
• Chronic hemodialysis	17 (5.0)	12 (3.9)	5 (18.5)	0.01
• Cirrhosis	13 (3.8)	13 (4.1)	0 (0)	0.61
Type of ICU admission, n (%)				0.95
• Medical	242 (71.6)	222 (71.4)	20 (74.1)	
• Emergency surgery	71 (21.0)	66 (21.2)	5 (18.5)	
• Planned surgery	25 (7.4)	23 (7.4)	2 (7.4)	
Reason for ICU admission, n (%)				0.07
• Cardiovascular failure	135 (39.9)	127 (40.8)	8 (29.6)	
• Infection	72 (21.3)	65 (20.9)	7 (25.9)	
• Neurological failure	29 (8.6)	23 (7.4)	6 (22.2)	
• Respiratory failure	70 (20.7)	65 (20.9)	5 (18.5)	
• Other	32 (9.5)	31 (10.0)	1 (3.7)	
SAPS II score on admission	54 [39–68]	54 [39–67]	57 [40–75]	0.49
SOFA score on admission	12 [9–14]	12 [9–14]	12 [9–15]	0.58
Mechanical ventilation on admission, n (%)	301 (89.1)	276 (88.7)	25 (92.6)	0.75
Length of hospital stay before ICU admission (days)	2 [0–6]	2 [0–6]	0 [0–5]	0.10
Hospitalization for more than 2 days within the past 6 months, n (%)	209 (61.8)	189 (60.8)	20 (74.1)	0.22
Use of indwelling catheter (urinary, gastric, tracheal), n (%)	3 (0.9)	3 (0.9)	0 (0)	1.00
Travel abroad in the last 6 months, n (%)	39 (11.5)	29 (9.3)	10 (37.0)	< 0.001
Antibiotic therapy in the 3 months prior to ICU-acquired infection				
• All types of antibiotic therapy, n (%)	269 (79.6)	246 (79.1)	23 (85.2)	0.62
o All types of β-lactam, n (%)	265 (78.4)	242 (77.8)	23 (85.2)	0.47
o β-lactam of class ≥3, n (%)	199 (58.9)	177 (56.9)	22 (81.5)	0.02
Class of β-lactam, n (%)				0.03
▪ Class 1	5 (1.5)	5 (1.6)	0 (0)	
▪ Class 2	61 (18.0)	60 (19.3)	1 (3.7)	
▪ Class 3	66 (19.5)	55 (17.7)	11 (40.7)	
▪ Class 4	80 (23.6)	75 (24.1)	5 (18.5)	
▪ Class 5	0 (0)	0 (0)	0 (0)	
▪ Class 6	53 (15.7)	47 (15.1)	6 (22.2)	
o Quinolone, n (%)	26 (7.7)	23 (7.4)	3 (11.1)	0.45
o Aminoglycoside, n (%)	120 (35.5)	108 (34.7)	12 (44.4)	0.40
o Vancomycin or linezolid, n (%)	47 (13.9)	40 (12.9)	7 (25.9)	0.08
Delay between prior antibiotic therapy and date of infection, n (%)				
o Between ICU admission and date of infection	219 (64.8)	203 (65.3)	16 (59.3)	0.54
o In the month prior to ICU admission	159 (47.0)	115 (37.0)	15 (55.5)	0.07
o Between 3 months and 1 month prior to ICU admission	19 (5.6)	13 (4.2)	2 (7.4)	0.34
Mechanical ventilation on the date of infection, n (%)	319 (94.4)	294 (94.5)	25 (92.6)	0.66
SOFA score on the date of infection	10 [6–13]	10 [6–13]	10 [6–12]	0.72
Delay between ICU admission and date of infection, (days)	7 [4–11]	6 [4–11]	9 [5–12]	0.06
• More than 5 days after admission, n (%)	239 (70.7)	216 (69.5)	23 (85.2)	0.12
• More than 7 days after admission, n (%)	173 (51.2)	154 (49.5)	19 (70.4)	0.05
Colonization with MDR/XDR at least 2 days before the date of infection, n (%)	55 (16.3)	41 (13.2)	14 (51.9)	< 0.001
Type of ICU-acquired infection, n (%)				0.02
• Bloodstream infection	140 (41.4)	123 (39.5)	17 (63.0)	
• Pneumonia	198 (58.8)	188 (60.5)	10 (37.0)	

*SAPS II* Simplified Acute Physiology Score II, *SOFA* Sequential Organ Failure Assessment

^a^Including any immunosuppressive diseases, hematologic diseases, treatment with immunosuppressive drugs within the previous 30 days, or corticosteroids at daily doses of at least 10 mg/day of a prednisone equivalent for more than 2 weeks, ^b^Selected threshold for chronic renal failure: Glomerular Filtration Rate (GFR) less than 60 ml/min/1.73 m^2^

### ICU-acquired infections

Our cohort of 338 patients with ICU-acquired infections included 140 cases of bloodstream infection and 198 cases of pneumonia. The 188 bacteria causing the 140 cases of bloodstream infection and the 271 bacteria causing the 198 cases of pneumonia are presented in Table 2, along with their susceptibility profile to β-lactams. All infections combined, 61.8% of the bacteria found were Enterobacteriaceae, 39.9% were non-fermenting Gram-Negative Bacilli (GNB), 6.5% were other GNB, and 27.5% were Gram-positive bacteria. Eighty-four percent of the ICU-acquired pneumonia cases in our cohort were identified by deep respiratory sampling (protected distal sampling or bronchoalveolar lavage), and 19.6% were identified by tracheobronchial aspiration. In addition, the median modified CPIS score was 7 [6–8] for the 198 pneumonia cases.

**Table 2:** Description of the bacteria causing ICU-acquired infection

Microorganism, n (%)	Total (*n* = 459)	Bloodstream infection (*n* = 188)	Pneumonia (*n* = 271)
Monomicrobial infections	233 (50.8)	101 (53.7)	132 (48.7)
Infections with two types of bacteria	85 (18.5)	30 (16.0)	55 (20.3)
Infections with three types of bacteria	20 (4.4)	9 (4.8)	11 (4.1)
Enterobacteriaceae	209 (45.5)	94 (50.0)	115 (42.4)
Resistance 0	159 (34.6)	67 (35.6)	92 (33.9)
Resistance 1	23 (5.0)	12 (6.4)	11 (4.1)
Resistance 2	24 (5.2)	15 (8.0)	9 (3.3)
Resistance 3	3 (0.7)	0 (0)	3 (1.1)
Non-fermenting Gram-negative bacteria	157 (34.2)	46 (24.5)	111 (41.0)
*Pseudomonas spp.* resistance 0	86 (18.7)	23 (12.2)	60 (22.1)
*Pseudomonas spp.* resistance 1	6 (1.3)	3 (1.6)	3 (1.1)
*Pseudomonas spp.* resistance 2	12 (2.6)	4 (2.1)	8 (3.0)
*Pseudomonas spp.* resistance 3	3 (0.7)	1 (0.5)	2 (0.7)
*Burkholderia cepacia*	4 (0.9)	0 (0)	4 (1.5)
*Stenotrophomonas maltophilia*	22 (4.8)	8 (4.3)	14 (5.2)
*Chryseobacterium spp*. resistance 2	1 (0.2)	0 (0)	1 (0.4)
*Chryseobacterium spp*. resistance 3	1 (0.2)	0 (0)	1 (0.4)
*Acinetobacter spp.* resistance 0	9 (2.0)	3 (1.6)	6 (2.2)
*Acinetobacter spp.* resistance 1	2 (0.4)	0 (0)	2 (0.7)
*Acinetobacter spp.* resistance 3	3 (0.7)	0 (0)	3 (1.1)
Other	8 (1.7)	1 (0.5)	7 (2.6)
Gram-positive bacteria	93 (20.3)	48 (25.5)	45 (16.6)
*Staphylococcus aureus spp. resistance* 0	34 (7.4)	7 (3.7)	27 (10.0)
*Staphylococcus aureus spp.* resistance 1	0 (0)	0 (0)	0 (0)
Coagulase-negative *Staphylococcus spp.* resistance 1	21 (4.6)	19 (10.1)	2 (0.7)
Coagulase-negative *Staphylococcus spp.* resistance 2	2 (0.4)	2 (1.1)	0 (0)
*Enterococcus spp.* resistance 0	15 (3.2)	15 (8.0)	0 (0)
*Enterococcus spp.* resistance 1	1 (0.2)	1 (0.5)	0 (0)
*Streptococcus spp.*	19 (4.1)	3 (1.6)	16 (5.9)
Other	1 (0.2)	1 (0.5)	0 (0)

Enterobacteriaceae resistance 0: cefotaxime-sensitive

Enterobacteriaceae resistance 1: cefotaxime-resistant and non-ESBL-producing

Enterobacteriaceae resistance 2: carbapenem-sensitive and ESBL-producing

Enterobacteriaceae resistance 3: carbapenem-resistant

*Pseudomonas spp.* resistance 0: ceftazidime-sensitive

*Pseudomonas spp.* resistance 1: ceftazidime-resistant and carbapenem-sensitive

*Pseudomonas spp.* resistance 2: carbapenem-resistant and ceftazidime-sensitive

*Pseudomonas spp.* resistance 3: ceftazidime- and carbapenem-resistant

Same classification for *Chryseobacterium spp.* and *Acinetobacter spp.*

*Staphylococcus spp.* resistance 0: oxacillin-sensitive

*Staphylococcus spp.* resistance 1: oxacillin-resistant and vancomycin-sensitive

*Staphylococcus spp.* resistance 2: oxacillin- and vancomycin-resistant

*Enterococcus spp.* resistance 0: ampicillin-sensitive

*Enterococcus spp.* resistance 1: ampicillin-sensitive and glycopeptide-resistant

*Enterococcus spp.* resistance 2: ampicillin- and glycopeptide-resistant

As regards microbial resistance, 7.1% of the strains were ESBL-E and 0.9% were carbapenem-resistant Enterobacteriaceae. Among non-fermenting GNB strains, 2.36% were resistant to ceftazidime and 8.6% were resistant to imipenem. A total of 48 bacteria were resistant to carbapenems, representing 14.20% of all ICU-acquired infections. Of these 48 strains, 41 were susceptible to a β-lactam of a lower class (including 12 *Pseudomonas* with resistance to imipenem).

### Colonization

In our cohort, 55 patients (16.3%) were colonized with MDR/XDR at least 2 days before the date of ICU-acquired infection. This status was known on ICU admission for 11 patients (3.2%). In the 55 patients colonized with MDR/XDR, the analysis found 81 bacterial strains, 68 of which were sensitive to carbapenems and 13 were resistant to carbapenems (Additional file 2: Table S2). There was a statistically significant association between colonization with MDR/XDR and “the presence of bacteria requiring carbapenem treatment” (*p* < 0.001).

### Empiric antibiotic therapy

Of the 342 antibiotic therapies administered to the 338 cohort patients, 40.6% were monotherapies and 44.7% were dual therapies (Table 3). The molecule most commonly administered as monotherapy was piperacillin-tazobactam (19.3%), followed by carbapenems (11.1%). Of all antibiotic therapies (whether monotherapies or multidrug therapies), 125 (36.5%) were empiric and included a carbapenem. Analysis of the adequacy of empiric antibiotic therapy with a carbapenem is detailed in Table 4. Antimicrobial susceptibility testing analysis of the infection-causing strains found that 68.8% of the 125 empiric antibiotic therapies with a carbapenem were ineffective. In addition, 20% of the patients who received empiric antibiotic therapy with a carbapenem had an infection with carbapenem-resistant bacteria.

**Table 3:** Description of the empiric antibiotic therapies administered to the 338 cohort patients

Administered class of antibiotics	Total (*n* = 342)	Bloodstream infection (*n* = 132)	Pneumonia (*n* = 206)
Monotherapy, n (%)	139 (40.6)	44 (33.3)	95 (46.1)
Amoxicillin-clavulanic acid	14 (4.1)	4 (3.0)	10 (4.9)
Third-generation cephalosporin	9 (2.6)	1 (0.8)	8 (3.9)
Fourth-generation cephalosporin	0 (0)	0 (0)	0 (0)
Piperacillin-tazobactam	66 (19.3)	23 (17.4)	43 (20.9)
Carbapenem	38 (11.1)	9 (6.8)	29 (14.1)
Vancomycin or linezolid	11 (3.2)	7 (5.0)	4 (1.9)
Dual therapy, n (%)	153 (44.7)	67 (50.8)	86 (41.7)
Amoxicillin-clavulanic acid and aminoglycoside	1 (0.3)	0 (0)	1 (0.5)
Amoxicillin-clavulanic acid and vancomycin	2 (0.6)	2 (1.5)	0 (0)
Third-generation cephalosporin and aminoglycoside	1 (0.3)	0 (0)	1 (0.5)
Fourth-generation cephalosporin and aminoglycoside	1 (0.3)	1 (0.8)	0 (0)
Piperacillin-tazobactam and aminoglycoside	64 (18.7)	20 (15.2)	44 (21.4)
Carbapenem and aminoglycoside	53 (15.5)	29 (22.0)	24 (11.7)
Piperacillin-tazobactam and vancomycin	8 (2.3)	4 (3.0)	4 (1.9)
Carbapenem and vancomycin	11 (3.2)	4 (3.0)	7 (3.4)
Other dual therapy	17 (5.0)	6 (4.5)	11 (5.4)
Combination of three or more antibiotics n (%)	49 (14.3)	23 (17.4)	26 (12.6)
Piperacillin-tazobactam, aminoglycoside, and vancomycin	16 (4.7)	8 (6.0)	8 (3.9)
Carbapenem, aminoglycoside, and vancomycin	23 (6.7)	13 (9.8)	10 (4.9)
Other combination	8 (2.3)	1 (0.8)	7 (3.4)
Change of antibiotics within 24 h of treatment introduction, n (%)	24 (7.0)	9 (6.8)	15 (7.3)

**Table 4:** Analysis of the efficacy of empiric antibiotic therapy with a carbapenem

Variable	Total (*n* = 125)	Bloodstream infection (*n* = 56)	Pneumonia (*n* = 69)
Appropriate choice of carbapenem, n (%)	14 (11.2)	5 (8.9)	9 (13.0)
Inappropriate choice of carbapenem, n (%)	111 (88.8)	51 (91.1)	60 (87.0)
• Overuse	86 (68.8)	45 (80.4)	41 (59.4)
• Failure	25 (20.0)	6 (10.7)	19 (27.5)

### Patient outcome in ICU

In total, 132 (39.1%) patients underwent surgical treatment, 151 (44.7%) received renal replacement therapy, and 79 (23.4%) received corticotherapy during their stay in ICU. For the total cohort of 338 patients, duration of mechanical ventilation and length of stay in ICU were 15 [9–25] and 17.5 [11–27] days, respectively. The in-ICU mortality rate was 31.1%.

### Subgroup analysis

Subgroup analysis according to type of ICU-acquired infection was conducted and is presented in Additional file 3: Table S3. In this subgroup analysis, the significant association found in the total cohort between “the presence of bacteria requiring carbapenem treatment” and “prior use of a β-lactam class ≥ 3” was no longer observed. Moreover, the statistical link for chronic renal failure was no longer found in the pneumonia subgroup. In the subgroup of bloodstream infection, “chronic hemodialysis”, “colonization with MDR/XDR bacteria known on admission” and “delay of more than 7 days between ICU admission and date of nosocomial infection” were no longer statistically associated with “the presence of bacteria requiring carbapenem treatment”.

### Multivariate logistic regression analysis and CarbaSCORE derivation

After multicollinearity analysis, variables collected at the time of empiric prescription of antibiotic therapy that were associated in bivariate analysis with “the presence of bacteria requiring carbapenem treatment” (with *p* < 0.05) were entered into a multivariate logistic regression analysis.

These variables were, namely, chronic hemodialysis, bloodstream infection, prior use of a β-lactam class ≥3, travel abroad in the last 6 months, MDR colonization and delay of more than 7 days between ICU admission and date of nosocomial infection.

The variable “chronic renal failure” was not selected due to collinearity with chronic hemodialysis. Since “MDR colonization” was strongly related to “use of a β-lactam class ≥ 3” (*p* < 0.001), and because we wanted a score that can be calculated in ICUs that do not analyze MDR colonization, we decided to enter a composite item in the model: “colonization with MDR bacteria at least 2 days before the date of ICU-acquired infection or use of a β-lactam class ≥ 3 (in the 3 months prior to infection)” in the same item of the score.

The independent variables selected in the final model are presented in Table 5. Thus, according to the chosen methodology, “presence of bloodstream infection” (as opposed to pneumonia) scored 2 points in the CarbaSCORE, “chronic hemodialysis” scored 4 points, “travel abroad in the last 6 months” scored 5 points and, finally, “colonization with MDR bacteria at least 2 days before the date of ICU-acquired infection or use of a β-lactam class ≥ 3 (in the 3 months prior to infection)” scored 6 points. The obtained CarbaSCORE thus varied from 0 to 17 points.

**Table 5:** Variables independently associated with “presence of bacteria requiring carbapenem treatment” in multivariate analysis

Variable	Adjusted Odds Ratios [CI 95%]	*p*	Points
Bloodstream infection	2.55 [1.05–6.18]	0.04	2
Chronic hemodialysis	3.44 [0.97–12.18]	0.056	4
Travel abroad in the last 6 months	4.02 [1.56–10.37]	0.004	5
MDR colonization or use of a β-lactam class ≥3 (in the 3 months prior to infection)	5.27 [2.17–12.81]	< 0.001	6

*CI* Confidence Interval. Hosmer-Lemeshow test value was *p* = 0.86. Nagelkerke and Cox/Snell *R*^2^ were 0.22 and 0.10, respectively

**Fig. 2:**
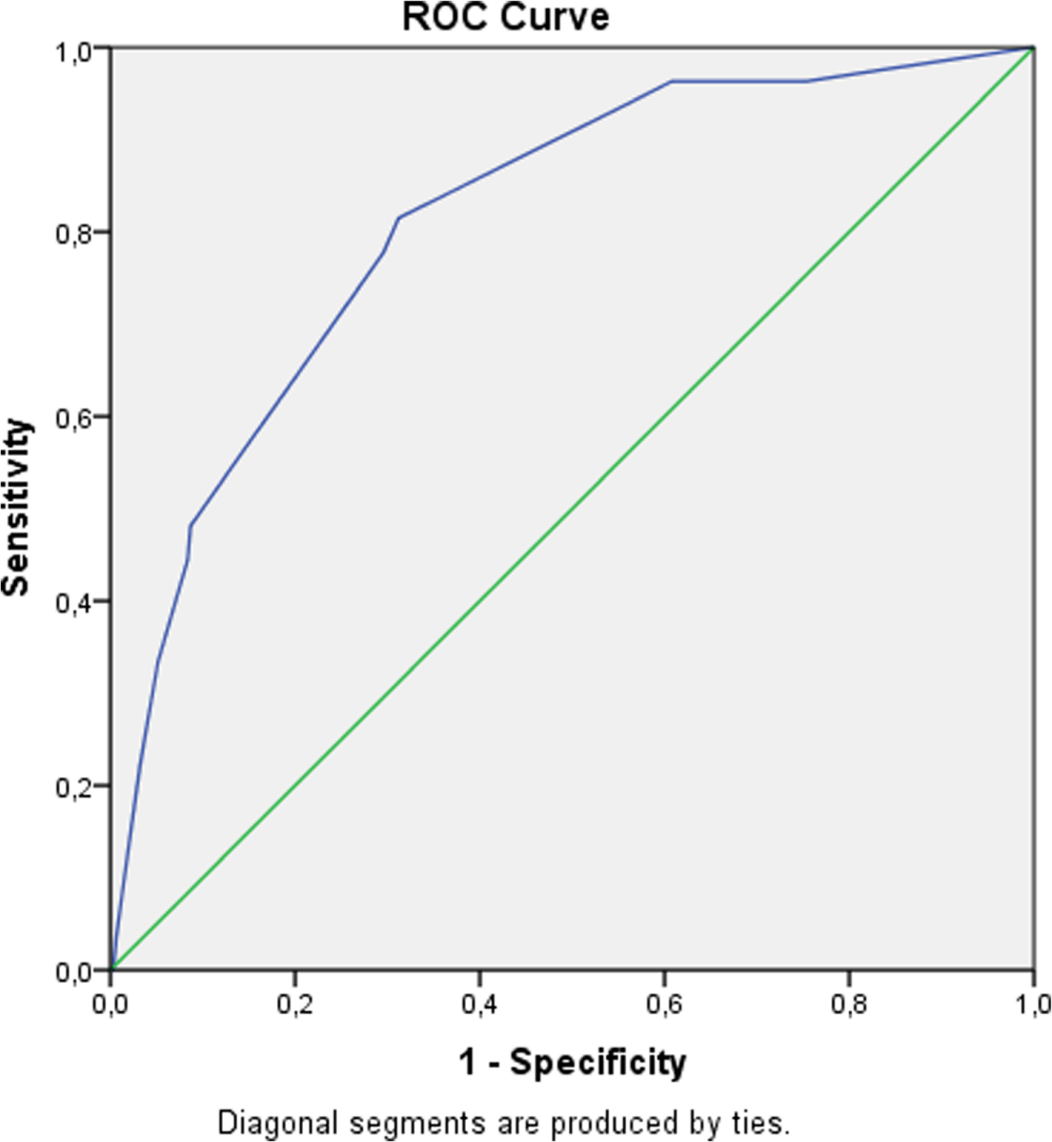
ROC curve of CarbaSCORE predicting a required carbapenem treatment in ICU-acquired pneumonia and bloodstream infection

Internal validation of this score by bootstrapping (2000 replicates) showed an area under the ROC curve of 0.81 [0.73–0.89]. The ROC curve obtained for the CarbaSCORE is shown in Fig. 2.

Table 6 presents the diagnostic performance of the CarbaSCORE in terms of sensitivity, specificity, positive and negative predictive values (with 95% Confidence Intervals determined by bootstrapping (2000 replicates)) for the main thresholds. We can see that sensitivity was 96 [89–100] % at the 6-point threshold and specificity was 91 [88–94] % at the 9-point threshold. The French recommendations of 2014 had a sensitivity and a specificity of 100 and 19%, respectively [19].

**Table 6:** Diagnostic performance of the CarbaSCORE to predict “the presence of bacteria requiring carbapenem treatment”

CarbaSCORE threshold	Sensitivity [CI 95%]	Specificity [CI 95%]	PPV [CI 95%]	NPV [CI 95%]
3	0.96 [0.89–1.00]	0.37 [0.32–0.42]	0.12 [0.11–0.13]	0.99 [0.97–1.00]
5	0.96 [0.89–1.00]	0.38 [0.33–0.43]	0.12 [0.11–0.13]	0.99 [0.97–1.00]
6	0.96 [0.89–1.00]	0.39 [0.34–0.45]	0.12 [0.11–0.13]	0.99 [0.97–1.00]
7	0.81 [0.67–0.96]	0.69 [0.64–0.74]	0.19 [0.15–0.22]	0.98 [0.96–1.00]
8	0.78 [0.63–0.93]	0.70 [0.66–0.76]	0.19 [0.15–0.23]	0.97 [0.96–0.99]
9	0.48 [0.30–0.67]	0.91 [0.88–0.94]	0.32 [0.21–0.46]	0.96 [0.94–0.97]
11	0.44 [0.26–0.63]	0.92 [0.88–0.95]	0.32 [0.20–0.45]	0.95 [0.93–0.97]
13	0.22 [0.07–0.41]	0.97 [0.95–0.98]	0.38 [0.15–0.62]	0.93 [0.92–0.95]

*PPV* Positive Predictive Value, *NPV* Negative Predictive Value, *CI* Confidence Interval

## Discussion

The ongoing problem of emerging MDR and XDR involves two risks: (*i*) the risk that empiric antibiotic therapy will fail in cases of nosocomial infection with resistant bacterial strains; and (*ii*) the risk that over-prescription of carbapenems will favor the development of XDR.

The aim of this study was to develop a simple score to help physicians prescribe carbapenems more sparingly in the treatment of major ICU-acquired infections, an issue that has not been well studied in the literature. The CarbaSCORE is a simple tool in that data for three of its four criteria are easily obtained from the patient. Data for the last criterion, “presence of bloodstream infection” (as opposed to pneumonia), are generally obtained from the clinical presentation or from laboratory results at the onset of ICU-acquired nosocomial infection. The diagnostic performance of the CarbaSCORE, which was validated in our cohort by bootstrapping, is interesting. On the one hand, a satisfactory sensitivity of 96 [89–100] % was achieved at the 6-point threshold, making it an appropriate choice for the most severe forms of infection. On the other hand, a specificity of 91 [88–94] % was achieved at the 9-point threshold, suggesting that it is a more appropriate choice for less severe forms. It should be noted that up to 2014, the French recommendations had a sensitivity and a specificity of 100 and 19%, respectively [19]. While updated recommendations propose taking into account case severity (acute respiratory distress syndrome, septic shock), the performance of the new criteria has not been assessed in the literature.

Two other scores have been proposed in the literature to address this issue, but they all have several major limitations. First, the score proposed by Vasudevan et al. in 2014 to predict a resistant GNB infection [34]. Definition of resistant GNB was problematic (*Acinetobacter baumannii*, *Pseudomonas aeruginosa*, *Klebsiella pneumoniae,* or *Escherichia coli* with resistance to one or more agents among at least three classes of antibiotics, without further details being provided). Moreover, the 76 patients with ICU-acquired resistant GNB infection were compared with 1398 patients with systemic inflammatory response syndrome criteria; and not with patients with non-resistant GNB infection. The validation cohort had an area under the ROC curve of 0.77[0.68–0.89]. Second, the score developed by Sonti et al. in 2017, was also aimed at predicting resistant bacterial infection in ICU patients, but the bacterial ecology reported in the study was composed of nearly 45% MRSA and GRE strains, making the score inapplicable in ICUs where the local bacterial ecology does not have this resistance profile. Moreover, the study did not include any internal or external validation [35]. Finally, these two scores included poorly defined items and the calculation of the score proposed by Vasudevan et al. was quite complex.

The cohort presented in our study was large given the topic studied. The most recent studies on this topic focused on smaller or similar patient cohorts [36–38]. For example, a recent study of risk factors for resistant GNB bloodstream infection in ICU used a cohort of 177 patients [36]. Our study population was a standard ICU population composed of patients aged 61 years on average and recruited from medical and surgical units. However, only documented infections were examined. Not only was the observed bacterial ecology similar to that found in other French studies on the topic, but it was very similar to the figures presented in the last national survey of prevalence in France [39]. The ecology of bacterial infections, such as those with a high prevalence of CPE, may be considerably different elsewhere in Europe or in the world, and the CarbaSCORE would probably not apply to them [7]. The rate of ICU-acquired infection (15.8%) found in our study was higher than the national average (10.4%), which may be explained by the severity profile of patients in our cohort: indeed, severity scores on admission were 54 for SAPS II and 12 for SOFA. The mortality rate (31.1%) and the high proportion of patients ventilated on admission (89.1%) also reflects the severity profile of our patients. Other factors associated with increased risk of infection should be emphasized: use of extracorporeal circulation techniques (cardiac surgery center, renal replacement therapy in 44.7% of patients), and cancer/immunocompromised status on admission (17.5% of patients). There was also a high proportion of patients with diabetes, which is a well-known risk factor for infection [40, 41].

Another interesting finding of our study is that more than one third of the treatments administered empirically included a carbapenem. However, antimicrobial susceptibility testing of the bacterial strains responsible for the infection revealed that only 11.2% of cases actually required carbapenem treatment. In a French observational study published in 2015, carbapenems were used three times out of four as empiric treatment for hospital-acquired infections; because this treatment seemed appropriate, it was continued in 21% of cases in 2009 and in 38% of cases in 2011 [42]. These observations confirm the interest of our study aimed precisely at improving empiric antibiotic therapy, given that the latter appears to perform poorly at the moment. Furthermore, empiric antibiotic therapy is not uncommon in cases where infection is not confirmed. It is therefore likely that overuse of carbapenems is even more important than suggested by the analysis of antibiotic therapies used to treat documented infections.

As regards risk factors for resistant bacterial infection, our results suggest that some of the criteria mentioned in national and international recommendations are of little interest. Although we did not observe the presence of acute respiratory distress syndrome or of septic shock, the SOFA score on the date of infection was not statistically associated with appropriate carbapenem prescription in bivariate analysis. We wish we had evaluated these two specific criteria, but the latest recommendations were published after the start of our study.

Similarly, the scientific basis for using prior colonization with MDR/XDR bacteria as a criterion to guide empiric antibiotic therapy seems questionable. For example, a 2013 meta-analysis focusing on 791 cases of VAP drawn from 13 studies found that infection-causing bacteria were effectively predicted by routine surveillance of the bacteria identified in deep respiratory samples. However, there appears to be an important bias, as colonization and infection were diagnosed by the same examination [43]. It should also be noted that practice recommendations do not include routine surveillance of respiratory ecology in the treatment of VAP [19, 38]. Moreover, a recent study investigating the predictive power of surveillance of rectal colonization with ESBL-E found a statistical link between rectal colonization with ESBL-E and the presence of ESBL-E in respiratory samples, with a sensitivity of 75.3[72.6–78.1] % and a specificity of 71[68.1–73.9] %. However, these samples were taken routinely, and not in the context of infection, thereby limiting the clinical relevance of the study [44].

In addition, most studies focusing on colonization with MDR bacteria as a criterion to guide empiric antibiotic therapy do not analyze the other risk factors for infection with MDR bacteria, thus raising the possibility of multicollinearities. Indeed, the analysis of our results yielded a strong collinearity between prior colonization with MDR/XDR bacteria and the variables “prior use of a β-lactam class ≥ 3”. Screening for colonization with MDR/XDR bacteria is not universal, and some authors even propose to abandon the practice. For example, a recent before-and-after study found that discontinuation of routine surveillance of colonization with ESBL-E in ICU (with continuation of recommended hygiene measures, including routine isolation of patients) was not associated with an increase in the incidence of ESBL-E infection (*p* = 0.64), although it was accompanied by a significant decrease in patient exposure to carbapenems during the stay in ICU (*p* = 0.01) [45].

Another strength of our study is that cases of bloodstream infection and pneumonia were analyzed globally so as to propose a single score for both types of infection. Moreover, we did not limit our analysis to infections caused by Enterobacteriaceae. Many studies focus exclusively on the analysis of risk factors for ESBL-E infection [46]; but in practice, when prescribing empiric antibiotic therapy, ICU physicians cannot know whether infections are caused by Enterobacteriaceae or, for example, by non-fermenting GNB.

Our study has many limitations. First, our methodology involves biases and limits extrapolation of results. Although we have shown that the demographic and microbiological characteristics of our patients were standard in France, this study remains a monocentric study with results that are difficult to extrapolate, especially where ecology is quite different. Also, the statistical power of the cohort was moderate, with 27 cases of ICU-acquired infection requiring carbapenem treatment. Second, in our methodology, treatment with a β-lactam class < 5 was considered as therapeutic failure in patients infected with ESBL-E, which can be discussed. Indeed, the literature shows that there are effective alternatives for the treatment of mild ESBL-E infections with no deep infection foci and if the inhibitory concentration (MIC) is low [45–48]. Lastly, we could not analyze some of the criteria proposed by the latest American and French recommendations for the management of hospital-acquired pneumonia, because our study was initiated before these recommendations were published (in 2016 and 2017, respectively). It should be noted, however, that the criteria “acute respiratory distress syndrome during pneumonia” and “septic shock during pneumonia” are not based on a solid review of the literature. In the same way, we did not evaluate the scores of Vasudevan and Sonti of on our cohort because of the many biases of these studies [34, 35]. Additional studies are therefore needed to assess the actual performance of the CarbaSCORE for all ICU-acquired infections, but also for infections acquired outside the ICU setting.

## Conclusions

Our study proposes the CarbaSCORE, which is aimed at helping ICU physicians prescribe carbapenems sparingly in the empiric antibiotic therapy of the first episode of ICU-acquired bloodstream infection or pneumonia. This score is composed of four simple criteria, and its accuracy is good. Moreover, the score varies from 0 to 17 points, which may allow for choosing a different threshold depending on case severity and on level of risk-taking. Additional studies, including validation studies, are required for the CarbaSCORE to be used in practice.

## Additional files

Additional file 1: **Table S1.** Classification of β-lactams following Weiss et al. (DOCX 12 kb)

Additional file 2: **Table S2.** Description of colonizing bacteria isolated in ICU. (DOCX 15 kb)

Additional file 3: **Table S3.** Sociodemographic, clinical-biological, therapeutic, and outcome characteristics of the 338 cohort patients: subgroup analysis. (DOCX 27 kb)

## Abbreviations

BSI: Bloodstream infection
CFU: Colony-forming units
CPE: Carbapenemase-producing Enterobacteriaceae
CPIS: The Clinical Pulmonary Infection Score
ESBL-E: Extended-spectrum β-lactamase-producing Enterobacteriaceae
GNB: Gram-Negative Bacilli
GRE: Glycopeptide-resistant enterococci
ICU: Intensive care unit
MDR: Multidrug-resistant bacteria
MIC: Minimum inhibitory concentration
MRSA: Methicillin-resistant *Staphylococcus aureus*
ROC: Receiver operating characteristic
SAPS II: Simplified Acute Physiology Score II
SFAR: Société Française d’Anesthésie et de Réanimation
SOFA: Sequential Organ Failure Assessment
VAP: Ventilator-associated pneumonia
XDR: Extensively Drug-Resistant
