# Supplementary material for: Derivation and validation of a simple score to predict the presence of bacteria requiring carbapenem treatment in ICU-acquired bloodstream infection and pneumonia: CarbaSCORE
Source: Antimicrob Resist Infect Control. 2019 May 20;8:78. doi: 10.1186/s13756-019-0529-z (PMC6528287; doi:10.1186/s13756-019-0529-z)
Supplement: Supplementary file 1 — Table S1. Classification of β-lactams following Weiss et al. (DOCX 12 kb) [file 13756_2019_529_MOESM1_ESM.docx]

**Table S1**. Classification of β-lactams following Weiss *et al.*

| Class | Molecule |
| --- | --- |
| 1 | Amoxicillin |
| 2 | Amoxicillin-clavulanic acid |
| 3 | Third-generation cephalosporin  Ureido/carboxypenicillin |
| 4 | Piperacillin-tazobactam  Ticarcillin-clavulanic acid  Fourth-generation cephalosporin  Anti-*Pseudomonas* third-generation cephalosporin |
| 5 | Ertapenem |
| 6 | Imipenem  Meropenem  Doripenem |
