# Supplementary material for: Derivation and validation of a simple score to predict the presence of bacteria requiring carbapenem treatment in ICU-acquired bloodstream infection and pneumonia: CarbaSCORE
Source: Antimicrob Resist Infect Control. 2019 May 20;8:78. doi: 10.1186/s13756-019-0529-z (PMC6528287; doi:10.1186/s13756-019-0529-z)
Supplement: Supplementary file 2 — Table S2. Description of colonizing bacteria isolated in ICU. (DOCX 15 kb) [file 13756_2019_529_MOESM2_ESM.docx]

**Table S2.** Description of colonizing MDR bacteria isolated in Intensive care unit

| Isolated microorganism, n (%) | Total (n=81) | Bacteremia (n=35) | Pneumonia (n=46) |
| --- | --- | --- | --- |
| ESBL-producing Enterobacteriaceae | 62 (76.5) | 28 (80.0) | 34 (73.9) |
| Carbapenem-resistant Enterobacteriaceae | 1 (1.2) | 1 (2.9) | 0 (0) |
| Carbapenemase-producing Enterobacteriaceae | 1 (1.2) | 0 (0) | 1 (2.2) |
| Ceftazidime-resistant *Pseudomonas spp.* | 4 (4.9) | 1 (2.9) | 3 (6.5) |
| Carbapenem-resistant *Pseudomonas* *spp.* | 3 (3.7) | 1 (2.9) | 2 (4.3) |
| Ceftazidime- and carbanapem-resistant *Pseudomonas* *spp.* | 0 (0) | 0 (0) | 0 (0) |
| Ceftazidime-resistant *Acinetobacter* *baumannii* | 2 (2.5) | 0 (0) | 2 (4.3) |
| Carbapenem-resistant *Acinetobacter* *baumannii* | 6 (7.4) | 3 (8.6) | 3 (6.5) |
| Methicillin-resistant *Staphylococcus aureus spp.* | 2 (2.5) | 1 (2.9) | 1 (2.2) |

ESBL: Extended-spectrum β-lactamase; MDR: MultiDrug Resistant
