# Supplementary material for: Derivation and validation of a simple score to predict the presence of bacteria requiring carbapenem treatment in ICU-acquired bloodstream infection and pneumonia: CarbaSCORE
Source: Antimicrob Resist Infect Control. 2019 May 20;8:78. doi: 10.1186/s13756-019-0529-z (PMC6528287; doi:10.1186/s13756-019-0529-z)
Supplement: Supplementary file 3 — Table S3. Sociodemographic, clinical-biological, therapeutic, and outcome characteristics of the 338 cohort patients: subgroup analysis. (DOCX 27 kb) [file 13756_2019_529_MOESM3_ESM.docx]

**Table S3.** Sociodemographic, clinical-biological, therapeutic, and outcome characteristics of the 338 cohort patients: subgroup analysis.

| Variable | Total  (n=338) | Bacteremia  (n=140) | | | Pneumonia  (n=198) | | |
| --- | --- | --- | --- | --- | --- | --- | --- |
|  |  | Carbapenem not necessary  (n=123) | Carbapenem necessary  (n=17) | *p* | Carbapenem not necessary  (n=188) | Carbapenem necessary  (n=10) | *p* |
| Age (years) | 61 [49-71] | 61 [51-70] | 69 [52-76] | 0.19 | 60 [46-71] | 64 [52-70] | 0.69 |
| Male sex, n (%) | 243 (71.9) | 87 (70.7) | 10 (58.8) | 0.40 | 137 (72.9) | 9 (90.0) | 0.46 |
| Chronic diseases, n (%) |  |  |  |  |  |  |  |
| - Chronic high blood pressure | 186 (55.0) | 68 (55.3) | 12 (70.6) | 0.30 | 100 (53.2) | 6 (60.0) | 0.75 |
| - Coronary artery disease | 85 (25.1) | 35 (28.5) | 6 (35.3) | 0.58 | 42 (22.3) | 2 (20.0) | 1.0 |
| - Arteriopathy | 59 (17.5) | 27 (22.0) | 4 (23.6) | 1.0 | 26 (13.8) | 2 (20.0) | 0.64 |
| - Diabetes | 117 (34.6) | 49 (39.8) | 6 (35.3) | 0.80 | 61 (32.4) | 1 (10.0) | 0.18 |
| - Chronic heart failure | 64 (18.9) | 17 (13.8) | 5 (29.4) | 0.15 | 40 (21.3) | 2 (20.0) | 1.0 |
| - Chronic respiratory failure | 46 (13.6) | 18 (14.6) | 2 (11.8) | 1.0 | 25 (13.3) | 1 (10.0) | 1.0 |
| - Chronic obstructive bronchitis | 46 (136) | 18 (14.6) | 3 (17.6) | 0.72 | 25 (13.3) | 0 (0) | 0.62 |
| - Cancer, immunosuppression | 59 (17.5) | 19 (15.4) | 3 (17.6) | 0.73 | 34 (18.1) | 3 (30.0) | 0.40 |
| - Chronic alcoholism | 98 (29.0) | 36 (29.3) | 5 (29.4) | 1.0 | 54 (28.7) | 3 (30.0) | 1.0 |
| - Chronic tobacco exposure | 91 (26.9) | 30 (24.4) | 4 (23.6) | 1.0 | 55 (29.3) | 2 (20.0) | 0.73 |
| - Obesity | 59 (17.5) | 25 (20.3) | 5 (29.4) | 0.36 | 28 (14.9) | 1 (10.0) | 1.0 |
| - Undernutrition | 47 (13.9) | 16 (13.0) | 1 (5.9) | 0.69 | 28 (14.9) | 2 (20.0) | 0.65 |
| - Chronic renal failure | 55 (16.3) | 19 (15.4) | 7 (41.2) | 0.02 | 27 (14.4) | 2 (20.0) | 0.64 |
| - Chronic hemodialysis | 17 (5.0) | 7 (5.7) | 3 (17.6) | 0.10 | 5 (2.7) | 2 (20.0) | 0.04 |
| - Cirrhosis | 13 (3.8) | 7 (5.7) | 0 (0) | 0.60 | 6 (3.2) | 0 (0) | 1.0 |
| Type of ICU admission, n (%) |  |  |  | 0.62 |  |  | 0.61 |
| - Medical | 242 (71.6) | 90 (73.2) | 12 (70.6) |  | 132 (70.2) | 8 (80.0) |  |
| - Emergency surgery | 71 (21.0) | 26 (21.1) | 3 (17.6) |  | 40 (21.3) | 2 (20.0) |  |
| - Planned surgery | 25 (7.4) | 7 (5.7) | 2 (11.8) |  | 16 (8.5) | 0 (0) |  |
| Reason for ICU admission, n (%) |  |  |  | 0.44 |  |  | 0.13 |
| - Cardiovascular failure | 135 (39.9) | 45 (36.6) | 6 (35.3) |  | 82 (43.6) | 2 (20.0) |  |
| - Severe infection | 72 (21.3) | 26 (21.1) | 4 (23.6) |  | 39 (20.7) | 3 (30.0) |  |
| - Neurological failure | 29 (8.6) | 7 (5.7) | 3 (17.6) |  | 16 (8.5) | 3 (30.0) |  |
| - Respiratory failure | 70 (20.7) | 32 (26.0) | 3 (17.6) |  | 33 (17.6) | 2 (20.0) |  |
| - Other | 32(9.5) | 13 (10.6) | 1 (5.9) |  | 18 (9.6) | 0 (0) |  |
| SAPS II score on admission | 54 [39-68] | 53 [36-65] | 60 [43-77] | 0.22 | 54 [40-69] | 55 [39-62] | 0.77 |
| SOFA score on admission | 12 [9-14] | 12 [9-14] | 12 [11-14] | 0.43 | 12 [9-14] | 9 [5-10] | 0.04 |
| Mechanical ventilation on admission, n (%) | 301 (89.1) | 109 (88.6) | 17 (100.0) | 0.22 | 167 (88.9) | 8 (80.0) | 0.33 |
| Length of hospital stay before ICU admission, (days) | 2 [0-6] | 2 [0-6] | 4 [0-7] | 0.67 | 1 [0-5] | 6 [2-13] | 0.05 |
| Hospitalization for more than two days within the past six months, n (%) | 209 (61.8) | 82 (66.7) | 13 (76.5) | 0.58 | 107 (56.9) | 7 (70.0) | 0.52 |
| Use of indwelling catheter (urinary, gastric, tracheal), n (%) | 3 (0.9) | 2 (1.6) | 0 (0) | 1.0 | 1 (0.5) | 0 (0) | 1.0 |
| Travel abroad in the last six months, n (%) | 39 (11.5) | 14 (11.4) | 6 (35.3) | 0.02 | 15 (8.0) | 4 (40.0) | 0.01 |
| Colonization with MDR, n (%) |  |  |  |  |  |  |  |
| - Known on ICU admission, n (%) | 11 (3.3) | 3 (2.4) | 1 (5.9) | 0.41 | 5 (2.7) | 2 (20.0) | 0.04 |
| - Known at least two days before the date of infection, n (%) | 55 (16.3) | 16 (13) | 8 (47.1) | 0.002 | 25 (13.3) | 6 (60) | 0.001 |
| - Delay between MDR colonization and date of infection, (days) | 7 [4 –11] | 7 [4–11] | 5 [4–7] | 0.32 | 6 [3–8] | 11 [8-14] | 0.04 |
| Antibiotic therapy in the three months prior to ICU-acquired infection |  |  |  |  |  |  |  |
| - All types of antibiotic therapy, n (%) | 269 (79.6) | 102 (82.9) | 15 (88.2) | 0.74 | 144 (76.6) | 8 (80.0) | 1.0 |
| - All types of β-lactam, n (%) | 265 (78.4) | 99 (80.5) | 15 (88.2) | 0.74 | 143 (76.1) | 8 (80.0) | 1.0 |
| - β-lactam of class ≥ 3, n (%) | 199 (58.9) | 77 (62.6) | 14 (82.4) | 0.17 | 100 (53.2) | 8 (80.0) | 0.12 |
| - Class of β-lactam, n (%) |  |  |  | 0.37 |  |  | 0.16 |
| - - Class 1 | 5 (1.5) | 2 (1.6) | 0 (0) |  | 3 (1.6) | 0 (0) |  |
| - - Class 2 | 61 (18.0) | 20 (16.3) | 1 (5.9) |  | 40 (21.3) | 0 (0) |  |
| - - Class 3 | 66 (19.5) | 23 (18.7) | 7 (41.2) |  | 32 (17.0) | 4 (40.0) |  |
| - - Class 4 | 80 (23.6) | 31 (25.2) | 4 (23.5) |  | 44 (23.4) | 1 (10.0) |  |
| - - Class 5 | 0 (0) | 0 (0) | 0 (0) |  | 0 (0) | 0 (0) |  |
| - - Class 6 | 53 (15.7) | 23 (18.7) | 3 (17.6) |  | 24 (12.7) | 3 (30.0) |  |
| - Quinolone, n (%) | 26 (7.7) | 12 (9.8) | 1 (5.9) | 1.0 | 11 (5.9) | 2 (20.0) | 0.13 |
| - Aminoglycoside, n (%) | 120 (35.5) | 37 (30.1) | 8 (47.1) | 0.17 | 71 (37.8) | 4 (40.0) | 1.0 |
| - Vancomycin or linezolid, n (%) | 47 (13.9) | 18 (14.6) | 4 (23.5) | 0.31 | 22 (11.7) | 3 (30.0) | 0.12 |
| - Delay between prior antibiotic therapy and date of ICU-acquired infection, n (%) |  |  |  |  |  |  |  |
| - - Between ICU admission and date of infection | 219 (64.8) | 89 (72.4) | 9 (52.9) | 0.16 | 114 (60.6) | 7 (70.0) | 0.74 |
| - In the month prior to ICU admission | 159 (47.0) | 49 (39.8) | 9 (52.9) | 0.31 | 66 (35.1) | 6 (60.0) | 0.17 |
| - Between three months and one month prior to ICU admission | 19 (5.6) | 7 (5.7) | 2 (11.8) | 0.3 | 6 (3.2) | 0 (0) | 1.0 |
| Mechanical ventilation on the date of infection, n (%) | 319 (94.4) | 109 (88.6) | 15 (88.2) | 1.0 | 185 (98.4) | 10 (100.0) | 1.0 |
| SOFA score on the date of infection | 10 [6-13] | 9 [6-12] | 9 [5-13] | 0.97 | 10 [7-13] | 10 [8-10] | 0.83 |
| Modified CPIS score on the date of pneumonia | NA | NA | NA | NA | 7 [6-8] | 8 [6-8] | 0.39 |
| Delay between ICU admission and date of infection, (days) | 7 [4-11] | 7 [4-11] | 8 [5-11] | 0.52 | 6 [3-10] | 11 [8-14] | 0.04 |
| - More than five days after admission, n (%) | 239 (70.7) | 92 (74.8) | 14 (82.4) | 0.76 | 124 (66.0) | 9 (90.0) | 0.17 |
| - More than seven days after admission, n (%) | 173 (51.2) | 64 (52.0) | 11 (64.7) | 0.44 | 90 (47.9) | 8 (80.0) | 0.06 |

SAPS II: Simplified Acute Physiology Score II. SOFA: Sequential Organ Failure Assessment. CPIS: Clinical Pulmonary Infection Score.
